# Obstetric Anal Sphincter Injuries: Risk Factors, Pelvic Floor Dysfunction, and Quality of Life Outcomes

**DOI:** 10.3390/medicina62030433

**Published:** 2026-02-25

**Authors:** Kristina Ivoskaite, Atene Simanauskaite, Egle Bartuseviciene, Dalia Regina Railaite, Laima Maleckiene, Justina Kacerauskiene

**Affiliations:** Faculty of Medicine, Medical Academy, Lithuanian University of Health Sciences, 44307 Kaunas, Lithuania

**Keywords:** OASIS, pelvic floor dysfunction, quality of life

## Abstract

*Background and Objectives*: Obstetric anal sphincter injuries (OASISs) are severe complications of vaginal delivery that can result in long-term pelvic floor dysfunction and reduced quality of life. Global data indicate a rising incidence of OASISs, including in Lithuania. This study aimed to identify risk factors for OASISs and evaluate their impact on urinary (UI) and fecal incontinence (FI), pelvic organ prolapse (POP), and quality of life in affected women. *Materials and Methods*: A retrospective case–control study was conducted at the Lithuanian University of Health Sciences Hospital (LUHS) Kauno Klinikos in 2024. Women who gave birth between 2004 and 2023 and experienced OASIS (*n* = 90) were compared with women matched for birth history but without perineal tears (*n* = 90). Data were collected from medical records and electronic questionnaires, including the International Consultation on Incontinence Questionnaire—Short Form (ICIQ-SF), Wexner score, Pelvic Organ Prolapse Symptom Score (POP-SS), and Pelvic Floor Impact Questionnaire (PFIQ-7). Participants were grouped by delivery year (2004–2013 or 2014–2023). Statistical analysis was performed using Mann–Whitney U, Chi-square, Fisher’s exact and Student’s *t*-tests, with *p* < 0.05 considered significant. *Results*: Newborn weight and vacuum-assisted delivery were significantly associated with OASIS (*p* < 0.05 and *p* = 0.029). In the 2014–2023 cohort, women with OASIS reported significantly higher rates and severity of UI, FI, and POP symptoms compared to controls. Quality of life scores related to UI and FI were significantly worse in the recent OASIS group, whereas no significant differences were observed in the 2004–2013 cohort. *Conclusions*: Between 2004 and 2023, 0.4% of women who gave birth at LUHS experienced third- or fourth-degree perineal tears, with newborn weight and vacuum extraction identified as risk factors. These women reported higher rates of UI and FI and POP, and those who delivered between 2014 and 2023 rated their related quality of life significantly worse than women without OASIS.

## 1. Introduction

Approximately 135 million births occur worldwide each year, and around 80% are vaginal deliveries [1,2]. Perineal trauma is common after vaginal birth, and while most tears are first- or second-degree, a clinically important proportion are severe. Obstetric anal sphincter injuries (OASISs) refer to third- and fourth-degree perineal tears that involve the anal sphincter complex, with fourth-degree tears additionally extending to the anal epithelium [3]. The reported OASIS incidence varies substantially across settings (approximately 0.5% to 11%), reflecting differences in obstetric practice, case mix, and ascertainment [4,5]. Over the past decade, increasing OASIS rates have been reported both in Lithuania and internationally (e.g., increases reported in the United Kingdom and Greece), highlighting the continuing relevance of prevention and long-term follow-up [3,6,7,8].

Importantly, an increase in reported OASIS rates does not necessarily indicate deterioration in care. Rising incidence can also reflect improved detection, standardized post-delivery assessment, and more accurate documentation and coding, particularly when routine structured examination is encouraged by professional guidance [9]. In parallel, prevention strategies have received growing attention, including standardized intrapartum perineal support (“hands-on” techniques) and appropriate use of lateral/mediolateral episiotomy in selected deliveries, with data suggesting that structured approaches can reduce OASIS rates in routine clinical practice [10,11].

Identifying risk factors is essential to guide prevention and counseling. OASIS risk factors are commonly grouped into maternal, fetal, and intrapartum factors. Maternal characteristics and labor management have been associated with OASIS risk, and delivery-related factors—especially operative vaginal delivery—are consistently highlighted as clinically important and potentially modifiable [4,12,13]. Recent evidence also underscores the potential for underdiagnosis of sphincter trauma, particularly involving the internal anal sphincter, which further supports careful assessment in higher-risk births and may influence the apparent incidence across institutions and time periods [14]. In vacuum-assisted births specifically, severe perineal lacerations have been associated with factors such as fetal position and intrapartum characteristics, reinforcing the importance of context-specific risk profiling [13].

Beyond the acute event and repair, OASIS can have long-term consequences for pelvic floor health. Women with OASIS are at increased risk of fecal incontinence, urinary incontinence, pelvic organ prolapse symptoms, and sexual dysfunction compared with women without OASIS, and these complications may present shortly after delivery or emerge years later [12,15,16]. Patient-reported outcomes are therefore critical to capture the broader symptom burden and quality-of-life impact. Evidence from long-term observational studies indicates that pelvic floor dysfunction relates to perineal tear severity and may persist beyond the early postpartum period [16], emphasizing the need for comprehensive follow-up across multiple pelvic floor domains rather than focusing solely on anal incontinence.

Despite the growing literature, evidence remains limited in some healthcare settings regarding how local risk profiles and evolving obstetric practice influence both OASIS occurrence and long-term pelvic floor outcomes assessed with validated questionnaires. Data from Lithuania on long-term patient-reported pelvic floor outcomes after OASIS remain limited, and local evidence is needed to inform counseling and prevention strategies in our healthcare setting. Comparing two delivery periods is particularly relevant because obstetric practice, training, and perineal assessment protocols may change over time, potentially affecting both true OASIS risk and ascertainment [9]. In addition, increased awareness of pelvic floor symptoms and access to follow-up care may influence long-term symptom reporting. Therefore, the objective of this study was to investigate risk factors contributing to OASIS and to evaluate long-term urinary and fecal incontinence, pelvic organ prolapse symptoms, and condition-specific quality of life using validated questionnaires in women with prior OASIS compared with controls, including a comparison between two delivery periods (2004–2013 vs. 2014–2023).

## 2. Materials and Methods

### 2.1. Study Design and Setting

A retrospective case–control study was conducted at the Department of Obstetrics and Gynecology, Lithuanian University of Health Sciences (LUHS), Kaunas Clinics. Routinely collected electronic medical records, birth records, and case histories were used to identify eligible women who delivered between 1 January 2004 and 31 December 2023. Data collection and participant contact were performed in 2024.

### 2.2. Participants, Eligibility Criteria, and Group Definition

Women were eligible for inclusion in the case group if they had a vaginal birth complicated by OASIS, defined as a third- or fourth-degree perineal tear. Records with missing key delivery information required for matching were excluded from matching and analysis.

### 2.3. Control Selection and Matching

For each woman with OASIS, one control (1:1 matching) was selected. Controls were defined as women who delivered vaginally during the same study period without any perineal tear. For each case, the control was selected as the next eligible woman who delivered in close temporal proximity and matched exactly on gravidity, parity, number of fetuses, and gestational age at delivery. Maternal age and pre-pregnancy body mass index (BMI), recognized risk factors for perineal trauma, were recorded and compared between groups; however, they were not used as strict matching criteria.

### 2.4. Data Sources and Variables

Using the hospital electronic database, women belonging to the case and to the control group were identified. Sociodemographic and anthropometric data, obstetrical history, and pregnancy- and delivery-related factors were extracted from medical records. Extracted variables included maternal age, gravidity and parity, pre-pregnancy body mass index (BMI), gestational age at delivery, neonatal birthweight, fetal position (occiput posterior, when documented), epidural analgesia, induction of labor, and vacuum extraction. Pre-pregnancy BMI was calculated based on self-reported or documented height and weight before pregnancy; obesity was defined as BMI ≥ 30 kg/m^2^. Data completeness of all variables was assessed. Records with missing matching values were excluded from matching, and remaining analyses were conducted using complete-case analysis.

### 2.5. Follow-Up and Patient-Reported Outcome Measures

**Figure 1:**
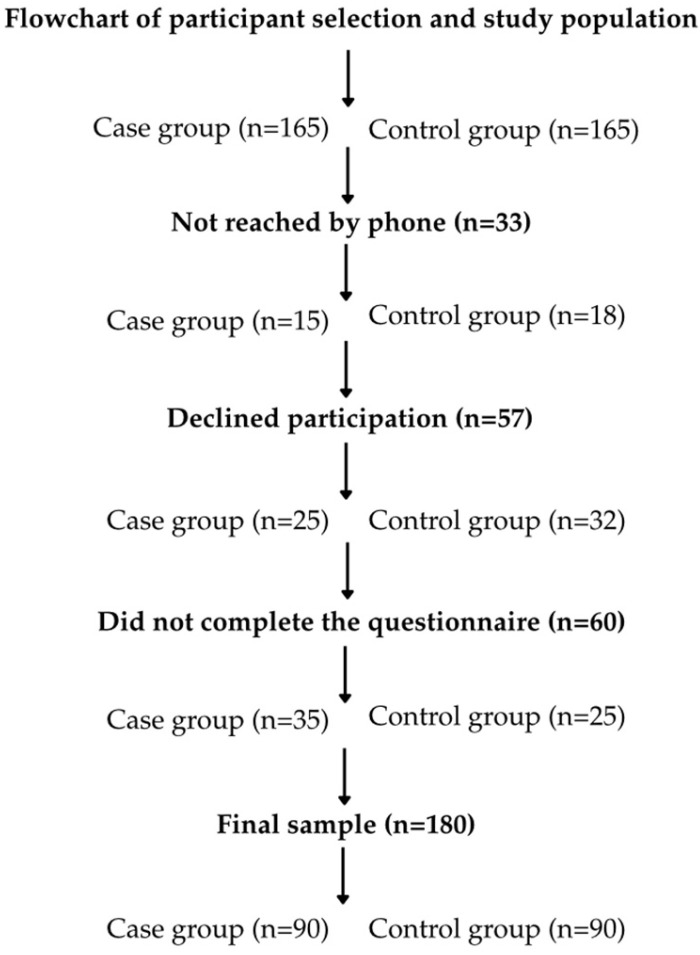
Flow diagram of participant identification, recruitment, and final study sample, including reasons for non-participation.

Eligible participants were contacted by telephone and invited to participate in the study. After giving verbal agreement, participants received an electronic questionnaire. Participant recruitment and reasons for non-participation are shown in Figure 1; some women could not be reached by phone, declined participation, or did not complete the questionnaire, resulting in a final sample of 180 women (90 cases and 90 controls). Due to the retrospective design of the study, the sample size was determined by the total number of eligible women with confirmed OASIS during the study period who met the inclusion criteria and agreed to participate. No formal a priori sample size calculation was performed. The survey included both researcher-developed questions and items from four validated instruments assessing long-term pelvic floor symptoms and quality-of-life impact: the International Consultation on Incontinence Questionnaire—Short Form (ICIQ-SF) for UI, total score 0–21, higher scores indicate more severe UI, the Wexner scale for FI, score 0–20, higher scores indicate more severe FI, the Pelvic Organ Prolapse Symptom Score (POP-SS) for POP symptoms, score 0–28, higher scores indicate more severe symptoms, and the Pelvic Floor Impact Questionnaire (PFIQ-7) for quality-of-life impact, score 0–300, higher scores indicate greater impact. All questionnaires were translated into Lithuanian and administered with standardized instructions.

### 2.6. Subgroup Definition by Delivery Period

To evaluate symptom burden and quality-of-life outcomes in relation to time since childbirth, participants were divided into two subgroups according to delivery period: 2004–2013 and 2014–2023. This grouping was chosen to reflect deliveries that occurred approximately 11–20 years prior to the study versus <10 years prior to the study. In the 2004–2013 subgroup, there were 20 women in each group (cases and controls), and in the 2014–2023 subgroup, there were 70 women in each group.

### 2.7. Ethical Approval

Approval for the study was obtained from the Kaunas Regional Biomedical Research Ethics Committee under the approval number BE–2–3. Data were anonymized prior to analysis.

### 2.8. Statistical Analysis

Anonymized data were organized and descriptive statistics were calculated using Microsoft Office Excel, and further analyses were performed using the statistical data analysis package IBM SPSS Statistics version 23. Categorical variables are presented as counts and percentages, and continuous variables as mean (standard deviation) or median (interquartile range), as appropriate. Group comparisons were performed using Student’s t-test for normally distributed continuous variables and the Mann–Whitney U test for non-normally distributed continuous variables. Associations between categorical variables were evaluated using the Chi-square (χ^2^) test or Fisher’s exact test. Results were considered statistically significant when *p* < 0.05.

## 3. Results

**Figure 2:**
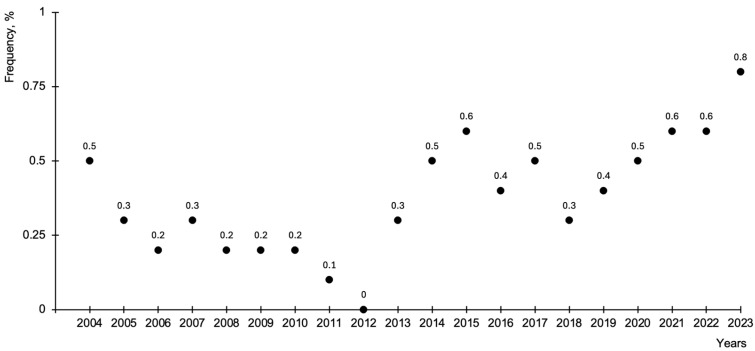
Frequency rates of OASIS from 2004 to 2023.

Between 1 January 2004 and 31 December 2023, a total of 45,433 women delivered vaginally at the Department of Obstetrics and Gynaecology of LUHS. OASISs were identified in 165 women, corresponding to an overall incidence of 0.4%. Of these cases, 47 occurred during the period 2004–2013, while 118 cases were recorded between 2014 and 2023, indicating a higher absolute number of OASIS diagnoses in the more recent decade (Figure 2).

Table 1 presents the sociodemographic characteristics of women in case and control groups. No statistically significant differences were observed between groups in terms of age, place of residence, education level, or marital status, suggesting that the groups were well matched and that these factors were unlikely to confound subsequent analyses.

Potential maternal, neonatal, and obstetric risk factors for OASIS are summarized in Table 2. Primiparity was common in both groups and did not differ significantly between cases and controls (63.3% in both groups; *p* = 1.000). Similarly, the prevalence of maternal obesity was higher in the case group but did not reach statistical significance (16.7% vs. 10.0%; *p* = 0.188).

In contrast, neonatal birthweight differed significantly between groups. Women who experienced OASIS delivered infants with a significantly higher mean birthweight compared with controls (3693.8 ± 370.9 g vs. 3332.0 ± 392.1 g; *p* < 0.001). Among obstetric factors, vacuum-assisted delivery was significantly more frequent in the case group (6.7%) than in the control group (0.0%; *p* = 0.029). No significant associations were observed for occiput posterior fetal position, induction of labor, or use of epidural analgesia.

UI before and after delivery was evaluated separately for the two delivery period cohorts (Table 3). Before delivery, UI was rare and did not differ significantly between groups in either period. After delivery, no significant difference in UI prevalence was observed between women with and without OASIS in the 2004–2013 cohort. However, in the 2014–2023 cohort, postpartum UI was reported significantly more frequently by women with OASIS compared with controls (45.7% vs. 25.7%; *p* = 0.014).

The severity, frequency, and volume of UI symptoms were assessed based on ICIQ-SF questionnaire scores and compared between groups using the Mann–Whitney U test. In the 2004–2013 cohort, no significant differences were detected between the case and control groups. In contrast, in the 2014–2023 cohort, the OASIS group demonstrated significantly higher ICIQ-SF scores, indicating more severe symptoms (Table 4).

FI symptoms were evaluated using the Wexner score (Table 5). In the earlier cohort (2004–2013), no statistically significant differences were observed between women with and without OASIS, either immediately after delivery or during follow-up. Conversely, in the 2014–2023 cohort, women with OASIS reported significantly higher Wexner scores both after delivery and at follow-up, reflecting greater severity and persistence of FI symptoms compared with controls (*p* < 0.001 for both comparisons).

POP symptoms were assessed using the POP-SS questionnaire (Table 6). No significant differences were identified between the case and control groups in the 2004–2013 cohort. However, in the 2014–2023 cohort, women with OASIS reported significantly higher POP-SS scores than women without perineal tears (*p* < 0.001), indicating a higher burden of prolapse-related symptoms in the more recent OASIS group.

Quality-of-life impact related to UI was assessed using the PFIQ-7 subscale (Table 7). In the 2004–2013 cohort, UI-related quality-of-life scores did not differ significantly between groups. In contrast, in the 2014–2023 cohort, women with OASIS reported a significantly greater negative impact on quality of life compared with controls (*p* = 0.035).

The Mann–Whitney U test was used to compare FI-related quality of life scores between women with OASIS (case group) and women without perineal tears (control group). In the 2004–2013 cohort, no significant difference was found between case and control groups. However, in the 2014–2023 cohort, although the median (Q1–Q3) FI-related quality-of-life scores were 0 (0–0) in both groups, the distribution differed, with higher ranks in the OASIS group (*p* = 0.047), indicating a significantly greater negative impact on quality of life compared with women who did not experience perineal tears (Table 8).

The Mann–Whitney U test showed no significant differences in evaluation of life quality related to POP between women in case and control groups in 2004–2013 and 2014–2023 (Table 9).

## 4. Discussion

Perineal tears after delivery, particularly those classified as OASIS, are associated with a higher risk of developing pelvic floor disorders later in life. These involve various conditions, such as UI, FI, POP, sexual dysfunction, and chronic pelvic pain [17]. Our study aimed to evaluate risk factors and long-term outcomes associated with OASIS following delivery.

Our findings confirm that instrumental delivery (in our study vacuum extraction) and fetal macrosomia significantly increase the risk of OASIS. These risk factors are well-established in the current literature and remain consistent across various populations and clinical settings [11,13,17]. Recent large-scale reviews and cohort studies underscore the role of instrumental delivery in OASIS. Similarly, Baruch et al. identified vacuum-assisted delivery as a significant risk factor for third- and fourth-degree tears (OR = 1.72), a pattern we observed in our cohort [11,13]. Moreover, Packet et al. emphasized the increased risk of OASIS in primiparous women undergoing instrumental birth, highlighting the importance of real-time sonographic assessment to detect underdiagnosed trauma. Their findings suggest that a considerable proportion of anal sphincter injuries may remain occult at the time of delivery, escaping detection by conventional clinical examination [17]. Similar observations demonstrated that up to one third of OASIS cases can be missed on routine postpartum inspection, but subsequently identified using endoanal ultrasound [14]. More recently, Guzmán Rojas et al. confirmed that systematic sonographic evaluation after instrumental delivery improves diagnostic accuracy and allows early intervention, thereby reducing the risk of long-term pelvic floor dysfunction [18]. Collectively, these findings highlight the need for standardized imaging protocols in high-risk deliveries, particularly among nulliparous women exposed to instrumental interventions. Targeted preventive strategies—such as perineal support and mediolateral episiotomy during operative births—can reduce the incidence of OASIS by more than 50% among nulliparous women [11].

While acute perineal trauma is well recognized, the long-term consequences for women’s quality of life are often underestimated [19,20]. Most available studies evaluate outcomes within the first months or up to one to two years after delivery, and robust data on the impact of OASIS 5, 10, or even 20 years later remain limited, leaving clinicians with little epidemiologic evidence to guide individualized counseling regarding prognosis, persistence, or progression of incontinence symptoms [21]. In our study, however, women with a history of OASIS exhibited significantly worse outcomes in domains related to incontinence, social participation, sexual function, and psychological well-being, even many years after childbirth. These findings are supported by Persson et al., whose prospective qualitative work from the post-OASIS project demonstrated that women who experienced OASIS continue to report persistent perineal pain and anal incontinence more than 18 months postpartum [22].

FI remains a predominant concern. Our results parallel those of Memon and Handa and more recent work from BMC Women’s Health, which found that women with OASIS score significantly worse on the Pelvic Floor Distress Inventory-20 (PFDI-20) and its Colorectal-Anal Distress Inventory-8 (CRADI-8) which measures distress from colorectal and anal symptoms and Pelvic Organ Prolapse Distress Inventory-6 (POPDI-6) that measures the distress caused specifically by POP symptoms subscales at both 3 and 12 months postpartum [19,20]. Chronic symptoms such as urgency to defecate, flatus incontinence, and soiling were associated with considerable social and emotional distress [22]. Importantly, epidemiologic estimates of incontinence prevalence may be inflated, as some studies classify women as incontinent even when functional control falls within normal limits. Furthermore, many symptomatic women never seek medical care—only approximately one quarter in the United States and one third in Europe consult a physician—which likely contributes to substantial under-recognition in clinical practice. Studies employing objective validation methods report significantly lower prevalence rates (23.5%) than those relying solely on interviews (29.5%), underscoring the challenges of accurately measuring disease burden [21]. These findings highlight the importance of integrating continence support and pelvic floor rehabilitation into postnatal care.

We observed that women evaluated in the later study period reported a notably higher prevalence of symptoms across all domains. This difference may partly reflect changing social and cultural attitudes toward postnatal health. Historically, women experiencing pelvic floor dysfunction after childbirth tended to accept symptoms as an unavoidable consequence of delivery, without seeking medical attention [23,24]. In contrast, more recent cohorts appear increasingly aware of pelvic floor health, more willing to discuss symptoms, and more likely to seek professional help. This trend is consistent with the broader increase in scientific and public attention to pelvic floor disorders [25,26]. A simple PubMed search illustrates this shift: during the earlier study period, only 25 publications addressed pelvic floor pathology (UI, FI, POP), whereas in the later period, 137 were identified. This substantial growth in research output likely reflects both improved recognition and heightened patient awareness [25].

This study has several limitations. The cross-sectional assessment of quality of life may not capture the full trajectory of recovery, and potential recall bias may have influenced self-reported outcomes. Moreover, our sample size may limit generalizability across diverse ethnic and socioeconomic populations. Future research should prioritize prospective, longitudinal studies with diverse cohorts to assess the effectiveness of specific interventions, including pelvic floor physical therapy, psychological counseling, and surgical repair techniques.

## 5. Conclusions

During study period 0.4% of women experienced OASIS. Newborn weight and the use of a vacuum extractor were identified as risk factors for this pathology. Women after OASIS reported UI, FI and POP symptoms more often after childbirth compared to those who did not experience tears. Furthermore, among women who gave birth between 2014 and 2023, those with OASIS rated their quality of life related to UI and FI worse than women without such injuries.

## Figures and Tables

**Table 1:** Sociodemographic data.

Criteria		Case Group	Control Group	*p*-Value
		*n* = 90	*n* = 90	
Patient age		28.99 ± 4.2 ^1^	29.17 ± 4.6 ^1^	0.787
Place of residence	Urban, *n* (%)	70 (77.8)	76 (84.4)	0.253
	Rural, *n* (%)	20 (22.2)	14 (15.6)	
Education	Primary, *n* (%)	5 (5.5)	0 (0.0)	0.384
	Secondary, *n* (%)	23 (25.6)	24 (26.7)	
	Higher, *n* (%)	62 (68.9)	66 (73.3)	
Marital status	Married, *n* (%)	72 (80.0)	66 (73.3)	0.379
	Partnership, *n* (%)	8 (8.9)	15 (16.7)	
	Single, *n* (%)	7 (7.8)	7 (7.8)	
	Divorced, *n* (%)	3 (3.3)	2 (2.2)	

^1^ Mean ± SD (SD, standard deviation).

**Table 2:** Risk factors for OASIS.

Risk Factor		Case Group	Control Group	*p*-Value
		*n* = 90	*n* = 90	
Maternal	Primipara, *n* (%)	57 (63.3)	57 (63.3)	1.000
	Obesity, *n* (%)	15 (16.7)	9 (10.0)	0.188
Newborn	Newborn weight, g	3693.8 ± 370.9 (2690–4915) ^1^	3332.0 ± 392.1 (1800–4205) ^1^	<0.001 ^2^
Obstetric	Occiput posterior position, *n* (%)	2 (2.2)	2 (2.2)	1.000
	Vacuum extraction, *n* (%)	6 (6.7)	0 (0.0)	0.029 ^3^
	Induction of labor, *n* (%)	19 (21.1)	21 (23.3)	0.720
	Epidural analgesia, *n* (%)	37 (41.1)	38 (42.2)	0.880

^1^ Mean ± SD (range), ^2^ Student’s *t*-test, ^3^ Fisher’s exact test.

**Table 3:** UI before and after delivery in OASIS and control groups.

Urinary Incontinence	Case Group (2004–2013)	Control Group (2004–2013)	*p*-Value	Case Group (2014–2023)	Control Group (2014–2023)	*p*-Value
	*n* = 20	*n* = 20		*n* = 70	*n* = 70	
Before delivery, *n* (%)	0 (0.0)	1 (5.0)	1.000	2 (2.9)	2 (2.9)	1.000
After delivery, *n* (%)	6 (30.0)	5 (25.0)	0.723	32 (45.7)	18 (25.7)	0.014 ^1^

^1^ Fisher’s exact test.

**Table 4:** ICIQ-SF score comparison between case and control groups.

Period	Case Median (Q1–Q3)	Control Median (Q1–Q3)	*p*-Value ^1^
2004–2013 (*n* = 20/20)	0 (0–6.5)	0.5 (0–7.5)	0.711
2014–2023 (*n* = 70/70)	0 (0–7)	0 (0–0)	0.047

^1^ Mann–Whitney U test.

**Table 5:** Comparison of Wexner score between women with and without OASIS.

Timepoint	Period	Case Median (Q1–Q3)	Control Median (Q1–Q3)	*p*-Value ^1^
After delivery	2004–2013 (*n* = 20/20)	0 (0–1.25)	0 (0–1)	0.960
	2014–2023 (*n* = 70/70)	2 (0–4)	0 (0–1)	<0.001
During follow-up	2004–2013 (*n* = 20/20)	0 (0–3.25)	0 (0–1)	0.593
	2014–2023 (*n* = 70/70)	1 (0–3)	0 (0–1)	<0.001

^1^ Mann–Whitney U test.

**Table 6:** Distribution of POP-SS scores in OASIS and control groups.

Period	Case Median (Q1–Q3)	Control Median (Q1–Q3)	*p*-Value ^1^
2004–2013 (*n* = 20/20)	3 (0.75–6)	3 (0.75–6.25)	0.967
2014–2023 (*n* = 70/70)	2 (0.25–7)	0 (0–2)	<0.001

^1^ Mann–Whitney U test.

**Table 7:** Comparison of quality of life impact of UI between OASIS and control groups.

Period	Case Median (Q1–Q3)	Control Median (Q1–Q3)	*p*-Value ^1^
2004–2013 (*n* = 20/20)	0 (0–2.25)	0 (0–1.5)	0.546
2014–2023 (*n* = 70/70)	0 (0–2)	0 (0–0)	0.035

^1^ Mann–Whitney U test.

**Table 8:** Effect of OASIS on quality of life due to FI.

Period	Case Median (Q1–Q3)	Control Median (Q1–Q3)	*p*-Value ^1^
2004–2013 (*n* = 20/20)	0 (0–0)	0 (0–0)	0.317
2014–2023 (*n* = 70/70)	0 (0–0)	0 (0–0)	0.047

^1^ Mann–Whitney U test.

**Table 9:** POP-related quality of life scores in women with and without OASIS.

Period	Case Median (Q1–Q3)	Control Median (Q1–Q3)	*p*-Value ^1^
2004–2013 (*n* = 20/20)	0 (0–0)	0 (0–0)	0.594
2014–2023 (*n* = 70/70)	0 (0–0)	0 (0–0)	0.101

^1^ Mann–Whitney U test.

## Data Availability

The original contributions presented in this study are included in the article. Further inquiries can be directed to the corresponding author.

## References

[1] United Nations, Department of Economic and Social Affairs, Population Division (2022). World Population Prospects: The 2022 Revision.

[2] World Health Organization (2021). Caesarean Section Rates Continue to Rise, Amid Growing Inequalities in Access.

[3] Frohlich J., Kettle C. (2015). Perineal care. BMJ Clin. Evid..

[4] Goh R., Goh D., Ellepola H. (2018). Perineal tears—A review. Aust. J. Gen. Pract..

[5] Ministry of Health of the Republic of Lithuania Guidelines (2019). Tarpvietės Plyšimai. Epiziotomija.

[6] Rodaki E., Diamanti A., Sarantaki A., Lykeridou A. (2022). The effects of perineal tears during childbirth on women’s sex life. Maedica.

[7] Tarasevičienė V., Biržietis T., Statnickaitė A., Andrijonytė M. (2013). Nėštumo ir gimdymo eigos įtaka III–IV laipsnio tarpvietės plyšimams. Lithuanian Obstetrics & Gynecology. 10-Asis Lietuvos Akušerių Ginekologų Draugijos Suvažiavimas: 2013 m. Rugsėjo 13-14 d., Šiauliai Lietuvos Akušerių Ginekologų Draugija—LAGD..

[8] Simanauskaitė A., Kačerauskienė J., Railaitė D.R., Bartusevičienė E. (2024). The Impact of Pelvic Floor Muscle Strengthening on the Functional State of Women Who Have Experienced OASIS After Childbirth. Medicina.

[9] (2015). The Management of Third- and Fourth-Degree Perineal Tears.

[10] Globerman D., Ramirez A.C., Larouche M., Pascali D., Dufour S., Giroux M. (2024). Guideline No. 457: Obstetrical Anal Sphincter Injuries (OASIS) Part I: Prevention, Recognition, and Immediate Management. J. Obstet. Gynaecol. Can..

[11] Eggebø T.M., Rygh A.B., von Brandis P., Skjeldestad F.E. (2024). Prevention of obstetric anal sphincter injuries with perineal support and lateral episiotomy: A historical cohort study. Acta Obstet Gynecol Scand..

[12] André K., Stuart A., Källén K. (2022). Obstetric anal sphincter injuries—Maternal, fetal and sociodemographic risk factors: A retrospective register-based study. Acta Obstet. Gynecol. Scand..

[13] Baruch Y., Gold R., Eisenberg H., Amir H., Yogev Y., Groutz A. (2022). Substantial Obstetric Anal Sphincter Injury during Vacuum-Assisted Delivery: An Obstetrical Issue or Device Related?. J. Clin. Med..

[14] O’Leary B.D., Kelly L., Fitzpatrick M., Keane D.P. (2023). Underdiagnosis of internal anal sphincter trauma following vaginal delivery. Ultrasound Obstet. Gynecol..

[15] Viannay P., De La Codre F., Brochard C., Thubert T., Meurette G., Legendre G., Venara A. (2021). Management and consequences of obstetrical anal sphincter injuries: Review. J. Visc. Surg..

[16] Huber M., Malers E., Tunón K. (2021). Pelvic floor dysfunction one year after first childbirth in relation to perineal tear severity. Sci. Rep..

[17] Packet M., Lefebvre G., Bujold E., Germain M., Plante L.A., Girard M. (2023). Prevalence and risk factors of sonographic obstetric anal sphincter injury (US-OASI): A systematic review and meta-analysis. Ultrasound Obstet. Gynecol..

[18] Guzmán Rojas R.A., Salvesen K.Å., Volløyhaug I. (2015). Anal sphincter defects and fecal incontinence 15–23 years after first delivery: A cohort study. Ultrasound Obstet. Gynecol..

[19] Walsh M., Fitzpatrick M., Anglim B. (2023). Obstetric anal sphincter injury—The long game: Primary and cumulative anal sphincter injury during childbirth and its long-term implications—A clinical practice review. Gynecol. Pelvic Med..

[20] Sartore A., Scalia M.S., Mangino F.P., Savastano G., Magni E., Ricci G. (2024). Pelvic floor function after third and fourth degree perineal lacerations: A case-control study on quality of life. BMC Womens Health.

[21] Bump R.C., Norton P.A. (1998). Epidemiology and natural history of pelvic floor dysfunction. Obstet. Gynecol. Clin. N. Am..

[22] Persson J., Lindqvist M., Stenholm S., Josefsson A. (2023). Long-Term Maternal Morbidity After Obstetric Anal Sphincter Injury (OASIS)—The POST-OASIS Project.

[23] Buurman M.B.R., Lagro-Janssen A.L.M. (2012). Women’s perception of postpartum pelvic floor dysfunction and their help-seeking behaviour: A qualitative interview study. Scand. J. Caring Sci..

[24] Lin J., Yu B., He Y., Tang N., He Q. (2025). Knowledge, attitude, and practice toward pelvic floor dysfunction among postpartum and postmenopausal women: A cross-sectional study. Int. Urogynecol. J..

[25] Martínez-Vázquez S., Peinado Molina R.A., Bermejo-Cantarero A., Martínez-Galiano J.M. (2024). A qualitative exploration of the perceptions of women living with pelvic floor disorders and factors related to quality of life. J. Clin. Med..

[26] Wu X., Yi X., Zheng X., Chen Z., Liu J., Dai X. (2023). Knowledge, attitudes, and practice of pelvic floor dysfunction and pelvic floor ultrasound among women of childbearing age in Sichuan, China. Front. Public Health.

